# Montreal Veterinary College; Commencement

**Published:** 1891-05

**Authors:** 


*McGill University. Faculty of Comparative Medicine and Veterinary Science; Commencement.*—The convocation of the Faculty of Comparative Medicine and Veterinary Science of McGill University, was held in the William Molson Hall, in the presence of a large audience. In the centre were the students of the various years and surrounding a number of ladies and their other friends.

At three o'clock Mr. J. W. Brakenridge, B. C. L., Registrar of the University, entered with the Chancellor, Sir Donald A. Smith; the Vice-Chancellor, Sir William Dawson; Dr. D. McEachran, dean of the Faculty of Comparative Medicine and Veterinary Science; the professors of the faculty and the members of the Convocation and the graduating class.

Among those who occupied seats on the platform were Dr. Stewart, of the medical faculty; Mr. J. J. Curran, Q.C., M. P., and Mr. John W. Gadsden, M. R. C. V. S., of Philadelphia.

Prof. McEachran, dean of the faculty, delivered a very short and pithy address. He pointed out that the accumulation of wealth marked the progress of a nation, and that McGill was rapidly becoming famous abroad. To what more useful purpose could the merchant princes put their money than to endow a seat of learning? McGill had much to be thankful for in this respect, but the Faculty of Comparative Medicine had been left to struggle alone.

Sir William Dawson, conferred the degree of V. S., on the following, in order of merit:

Geo. E. Macaulay, T. C. Simpson, T. B. McDonald, C. M. Higginson, S. S. Twombly, A. W. Gorham, D. B. Comstock, D. St. Louis, John Watson, Frank Barton, D. McDonald, Geo. A. Miller, John A. McCrank, Geo. Townsend.

Prizes were awarded as follows:

Veterinary Medicine and Surgery, George E. Macaulay; Anatomy, Thomas E. Simpson; Diseases of Cattle, D. B. Comstock; Chemistry, J. D. MacIntyre; Physiology, J. D. MacIntyre; Histology, Wilfrid Plaskett; Materia Medica, J. D. MacIntyre; Botany, Wilfrid Plaskett; Zoology, M. C. Wylie. For the best general examination on all subjects, (silver medal), Sidney S. Twombly; Second prize, (book), John A. McCrank.

The following gentlemen were awarded the degree as graduates of the late Montreal Veterinary College, who have been found to have complied with the regulations governing the granting of the degree to such;—

IN ABSENTIA.

Paul Paquin, Bacteriologist and Professor of Veterinary Science, Agricultural College, Columbia, Mo.; A. R. Rowat, Chief Veterinarian to the Government of Honolulu, S. I.; Peter Cummings, Lecturer of Anatomy, Quebec Veterinary College; John Robertson, Veterinary Surgeon, 2nd. U.S. Cavalry; John Ryan, Lecturer Chicago Veterinary College, Chicago, Ill., U. S.; Charles R. Simpson, Charlestown, Mass.; James B. Paige, Lecturer on Veterinary Medicine, Amherst Agricultural College, Amherst, Mass.; Archibald A. Keys, Minneapolis, Minn., U. S.; and Edward C. Crevier, Peterborough, Ontario.

After the Valedictory by Dr. McCrank, Dr. John W. Gadsden, of Philadelphia, was introduced by Sir Donald Smith, as being the first gentleman to make a valuable donation to the Faculty. Mr. Gadsden said he had watched the program of the Veterinary College for six years and was fully aware of the ability of the teaching staff of the college. Having collected a library, inclueing a complete set of the Veterinarian, and a museum and intending to retire from active life, he was glad as an Englishman to donate them to the Faculty, and he expressed the hope that it will receive endowments that will enable it to keep pace with the other faculties.

Mr. J. J. Curran, M. P., delivered an address to the graduating class. Sir William Dawson in making the closing remarks expressed himself as being heartily in sympathy with the work of the Comparative Medicine Faculty, but one thing he did not like was the present premises occupied by the Faculty. He hoped that in the near future they would be able to secure more commodious class-rooms and laboratories, and a hospital for the treatment of animals. His remarks were warmly applauded by the students present.

